# Investigation of the Pulsing Characteristic of a Carbon Nanotube Emitter

**DOI:** 10.3390/nano12030522

**Published:** 2022-02-02

**Authors:** Helin Zhu, Jejin Jang, Gyuwan Im, Hyungsoo Mok, Jehwang Ryu, Kyung-Seo Kim

**Affiliations:** 1 Department of Electrical Engineering, Konkuk University, Seoul 05029, Korea; wngkr3388@konkuk.ac.kr (H.Z.); kjk5412@konkuk.ac.kr (J.J.); gyuwanim@konkuk.ac.kr (G.I.); 2 Department of Physics, Kyung Hee University, Seoul 02453, Korea; jhryu@khu.ac.kr; 3 Department of Electrical Engineering, Sangmyung University, Seoul 110743, Korea; kskim@smu.ac.kr

**Keywords:** carbon nanotube, field emission, MOSFET, pulse drive, boosted current emission, pulse modulation

## Abstract

The carbon nanotube (CNT) field emitter is suitable for the high frequency pulsing of X-ray. Pulsing reduces 49% of the dose in grid-controlled fluoroscopy and improves the image of moving objects. Various structures and manufacturing processes are being studied. However, more studies on the dynamic characteristic of a pulsing CNT and its application are needed. In this study, the combined dynamics including the field emission, MOSFET, and modified gate driver for MOSFET have been analyzed. In this configuration, between the cathode of the tube and ground, there is a MOSFET switch that turns the tube current on/off and a shunt resistor that measures the tube current. Due to the high impedance of the vacuum between the gate and cathode of the tube, about 85% of the gate voltage is still exerted between the Gate and cathode of the tube during the off-state of the MOSFET. Therefore, space charges are built during the off-state and then released at the beginning of the on-state of the MOSFET. The modified gate driver structure for MOSFET that we propose in this paper can limit the amount of current flow through the cathode. Tube current (boosted current) can be accurately controlled through a modified gate driver structure. Combining the boosted current and pulse control of MOSFET, the dynamic current performance of a CNT tube can be enhanced and the average tube current or dose can be accurately controlled. Experiments, simulation, and analysis have been conducted to study the combined dynamics and its applications.

## 1. Introduction

Since Roentgen discovered X-ray with vacuum tubes, this spectrum of electromagnetic wave has been widely used in health care, industry, science, and many other fields [1,2,3,4]. The classical way of generating X-ray is to accelerate free electrons which are generated by the heating tungsten filament inside a vacuum tube by exerting high voltage [5,6,7]. The accelerated electrons then collide with anode to convert its tiny (typically about 1%) portion of kinetic energy to X-ray by bremsstrahlung [8]. 

As two key parts of X-ray technology, methods of generating high voltage and free electrons are improving alongside technological innovation. Power electronic technology allows a high voltage generator to exert fast and stable voltage across the anode and cathode of an X-ray tube with a relatively compact size [9]. The carbon nanotube (CNT)-based field emission allows the fast electron extraction of the cathode through electric energy rather than the thermal energy used in tungsten filaments, which has a slow dynamic characteristic [10,11,12]. 

The X-ray pulsing technique reduces up to 49% of the dose in grid-controlled fluoroscopy [13,14]. A pulse width of 5–10 ms allows for monitoring the respiratory and cardiac cycles and triggering imaging only at the desired physiologic phase [15]. X-ray with a pulse width less than 50 μs can be used to image a rotating cooling fan [16]. 

In recent years, research on CNT-based X-ray tubes has mainly focused on the microscopic structures of carbon nanotubes and their effects on performance, or how their manufacturing process associates with stability, longevity, and robustness [16]. The studies of current output characteristics (free electron extraction performance) are mainly focused on static or continuous applications [15]. The authors of [17] showed the X-ray pulse method using fast switching MOSFET between the cathode and ground. This method prevails in fast X-ray pulsing with CNT-based X-ray tubes. However, the current output characteristic under this configuration can be complicated by including MOSFET characteristics [18,19]. The properties of MOSFET could affect the pulsing dynamic. Recently, various novel materials and methods have been used to improve or modify the properties of MOSFET by manipulating electronic band structures between materials [20,21]. Applying these changes in properties of MOSFET could also affect the pulsing dynamic. It is necessary to investigate both the field emission characteristic and basic MOSFET switching dynamics. We refer to it as the combined dynamics of CNT pulsed X-ray. Studies on combined dynamics allow us to utilize it to enhance the performance of CNT based X-ray tubes.

In this paper, we present an improved pulsing method of a CNT-based X-ray generator. By studying the pulsing dynamic of X-ray through experiments, the influences of the CNT field emission curve and MOSFET switching mechanism are analyzed. For that, the gate driver circuit needed to be modified. The study of the combined dynamics of the field emission, MOSFET switching dynamics, and modified gate driver circuit is proposed in this paper. The basic equations of the field emission, MOSFET, and circuit are listed for analysis. Experiments and simulation are carried out to prove the claim. Finally, the improved method of utilizing the combined dynamics is presented. The improved pulsing method can boost its current output capability under the pulsing mode. With the current limiting function of the modified gate driver, dose accuracy can be enhanced as well.

## 2. Materials and Methods

**Figure 1:**
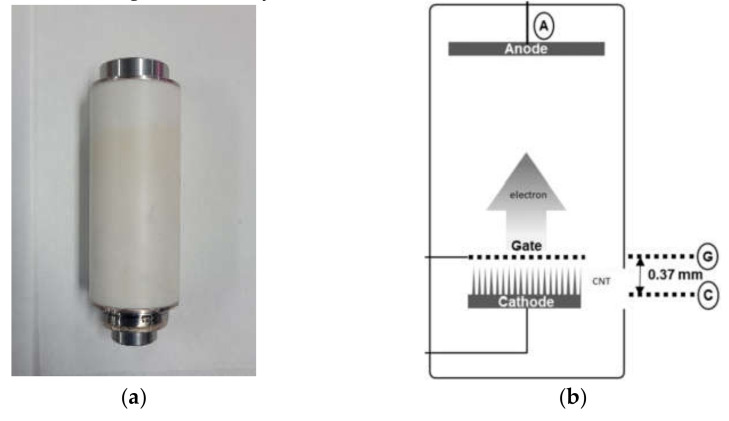
The CNT X-ray tube studied in this paper and its internal structure: (**a**) CNT tube, specification of 120 kV and 10 mA; (**b**) internal structure.

We used a typical CNT X-ray tube manufactured by Korean tube manufacturer CAT Beam Tech. The tube has the specifications of a tube voltage of 120 kV and cathode current of 6mA. The tube has a typical structure of three electrodes: anode, gate, and cathode as shown in Figure 1. The electrodes are sealed within a ceramic body. Between the metal electrode and ceramic body, a brazing process has been completed to bond two materials, keeping the internal vacuum. Higher than 1.5 kV of voltage across the gate and cathode can extract free electron form CNT. Up to 120 kV of voltage can accelerate free electrons towards the anode to generate X-ray. 

To study the details about the dynamics of CNT field emission under a pulsing condition using MOSFET, an experimental environment was set. A 5 kV power supply (Stanford research PS350) was used to drive the CNT gate. A high voltage power supply (Spellman SL300) was used to drive the anode. A function generator (Tektronix AFG 3022) was used to drive the MOSFET gate. The exerted tube gate voltage was 2.5–2.7 kV. The MOSFET gate was powered by a pulse of 10 V amplitude, 12.5–62.5 kHz frequency, and 50% duty cycle. IXTH02N450HV MOSFET was chosen to drive the pulse current. The shunt resistor of 500 Ω was used to monitor the pulse current by using an oscilloscope (LeCroy Wave Surfer 64Xs-A). Though the shunt resistor is meant to be just a current detector, the function of the resistor is essential in this layout and plays an important role to the combined characteristic of CNT X-ray pulsing.

**Figure 2:**
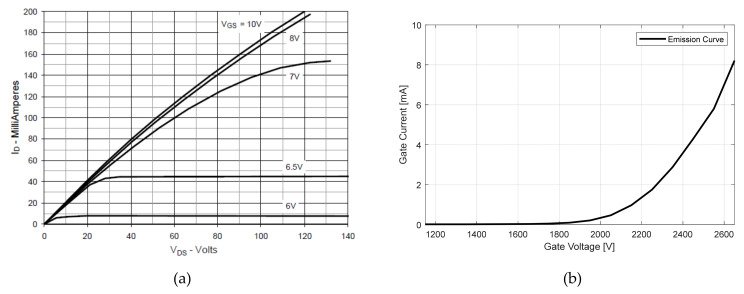
(**a**) The gate-source voltage vs drain current under drain-source voltage curve of IXTH02N450HV: by applying sufficient voltage (over 8 V), the MOSFET is fully turned on. The gate-source voltage must keep a stable value to ensure the on-state of the MOSFET; (**b**) measured emission curve of the CNT tube: the turn-on voltage is about 1800 V.

**Figure 3:**
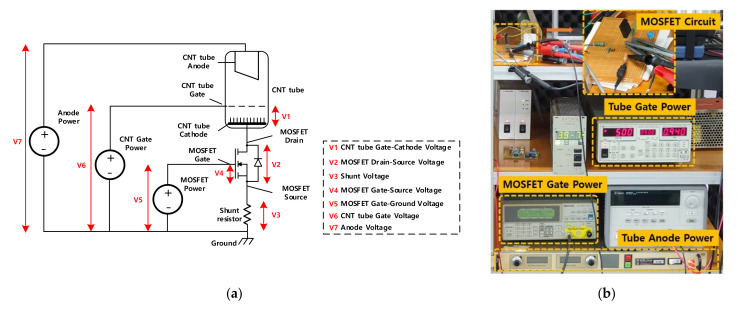
The experimental layout to study the dynamics of pulsing X-ray: (**a**) Three power sources— anode power, CNT gate power, and MOSFET power—drive the anode, CNT gate, and MOSFET gate, respectively. Every critical node of the layout is listed, and every important voltage is marked for analysis and discussion; (**b**) Experimental layout.

Figure 2 shows the switching characteristic curve of high voltage MOSFET from the datasheet of IXTH02N450HV. To maintain the on-state of the MOSFET, sufficient gate-source voltage for the MOSFET must be supplied (normally over 8 V). In this case, the specification of the tube current was about 8 mA. A gate-source voltage over 6 V is sufficient according to Figure 2. However, in the case of Figure 3, it is obvious that the gate-source voltage could vary by the tube current. In this layout, the design of the gate driver circuit of MOSFET is different from that in power electronics. Usually, the gate driver circuit should exert voltage between the MOSFET gate and source to supply a sufficient charging current [22,23]. In Figure 3, the gate driver circuit exerts voltage between the MOSFET gate and ground. The shunt resistor functions as feedback of the tube current (or drain current).

(1) $$V_{GS}=V_{GG}-R_{shunt}*I_{shunt}$$

**Figure 4:**
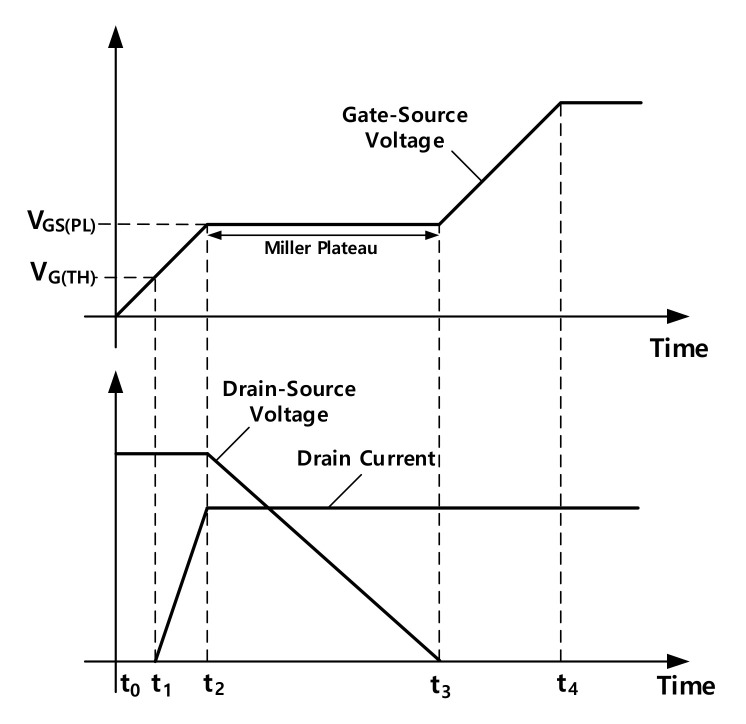
The turn on process of a typical N channel MOSFET: drain current increases from $t_{1}$ to $t_{2}$ and then reaches its maximum. The range from $V_{G\left(\mathrm{TH}\right)}$ to $V_{\mathrm{GS}\left(\mathrm{PL}\right)}$ is the channel opening period from the drain current perspective; drain-source voltage decreases from $t_{2}$ to $t_{3}$ and then reaches its minimum. The Miller plateau is the channel opening period from the drain-source voltage perspective.

Equation (1) shows the feedback function of the shunt resistor. Here, $V_{\mathrm{GS}}$ represents the gate-source voltage, $V_{\mathrm{GG}}$ represents the MOSFET power voltage, and $I_{\mathrm{shunt}}$ represents the current flow through the shunt resistor. When MOSFET is in the on-state, the current flows through the shunt resistor. This current causes a voltage increase of V3 which forms a negative feed to the gate-source voltage of the MOSFET, V4. As $I_{\mathrm{shunt}}$ gets higher, the $V_{\mathrm{GS}}$ gets lower, all the way to the threshold voltage of MOSFET which is described in Figure 4.

Figure 4 illustrates a typical switch-on period of N channel MOSFET. In Figure 4, $V_{G\left(\mathrm{TH}\right)}$ represents the threshold voltage of MOSFET. MOSFET will turn on when the gate-source voltage surpasses $V_{G\left(\mathrm{TH}\right)}$. $V_{\mathrm{GS}\left(\mathrm{PL}\right)}$ is the voltage level at which MOSFET enters a Miller plateau. The drain current increases from $t_{1}$ to $t_{2}$ and then reaches its maximum. Meanwhile, the drain-source voltage does not change in this period. The drain-source voltage decreases from $t_{2}$ to $t_{3}$ and then reaches its minimum. From the drain current perspective, the range from $V_{G\left(\mathrm{TH}\right)}$ to $V_{\mathrm{GS}\left(\mathrm{PL}\right)}$ is the channel opening period, which is important in our CNT pulsing technique.

The threshold voltage $V_{G\left(\mathrm{TH}\right)}$ determines the on-/off-state of MOSFET. If the shunt current rises, V3 increases as well, then as V4 drops below $V_{G\left(\mathrm{TH}\right)}$, the current will decrease. The decreased V4 will allow V3 to increase above the threshold voltage $V_{G\left(\mathrm{TH}\right)}$. Once negative feedback forms, the shunt current will stay at a critical value where V3 and V4 are balanced. The critical value determines the maximum current allowed for MOSFET in this layout. Equation (2) is the representation of the maximum current. The threshold voltage $V_{G\left(\mathrm{TH}\right)}$ is usually given by the MOSFET manufacturer and the value is fixed. The value in this case is about 5 V. 


(2) $$V_{\mathrm{GS}}={\;V}_{\mathrm{GG}}-R_{\mathrm{shunt}\;}{*\;I}_{\mathrm{shunt}}$$

## 3. Experiments

**Figure 5:**
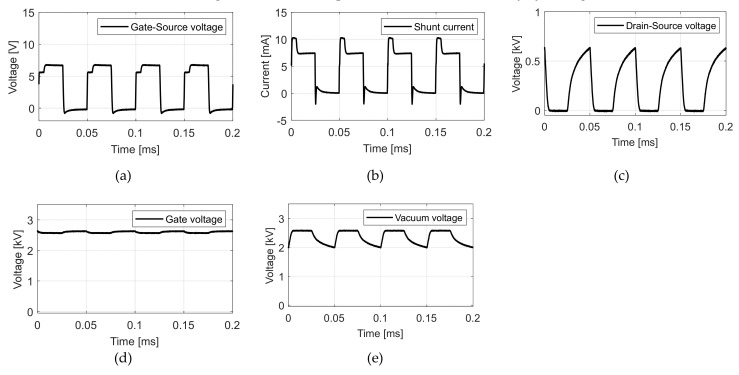
Experimental result of X-ray pulsing: (**a**) gate-source voltage of MOSFET; (**b**) shunt current; (**c**) drain-source voltage of MOSFET; (**d**) CNT gate voltage; (**e**) CNT gate-cathode voltage which causes field emission.

To verify the proposition, the experiment shown in Figure 3 was carried out. The anode voltage was 50 kV DC, the tube gate voltage was 2.6 kV DC, and the MOSFET gate voltage was 10 V with a pulse of 20 kHz and 50% duty cycle. Figure 5 shows the result. 

Here, Figure 5a represents the gate-source voltage of MOSFET. Figure 5b represents the shunt current. We can see that instead of a single pulse, two distinct levels of pulses appear at each turn on cycle of the MOSFET. To distinguish them, we call the higher level of the curve “high flat”, the lower level of the curve ”low flat”. The high flat current is about 10 mA, consisting of a boosted current at 2.5 mA and a fundamental current at 7.5 mA. The boosted current will be explained. For now, we can prove the claim about Equation (2), which says the maximum current is reached when V4 is close to $V_{G\left(\mathrm{TH}\right)}$ (5 V in this case). The high flat is the maximum current, and the low flat is the normal current result of the field emission. However, the appearance of high flat and low flat is not something that is normally expected to be seen for X-ray pulsing. To understand this phenomenon, further investigation and analysis were carried out. Figure 5c represents the drain-source voltage of MOSFET; Figure 5d represents the CNT gate voltage; Figure 5e shows the CNT gate-cathode voltage that causes normal field emission. It is also called vacuum voltage. The normal field emission we described here contributes to the fundamental current. 

## 4. Discussion

**Figure 6:**
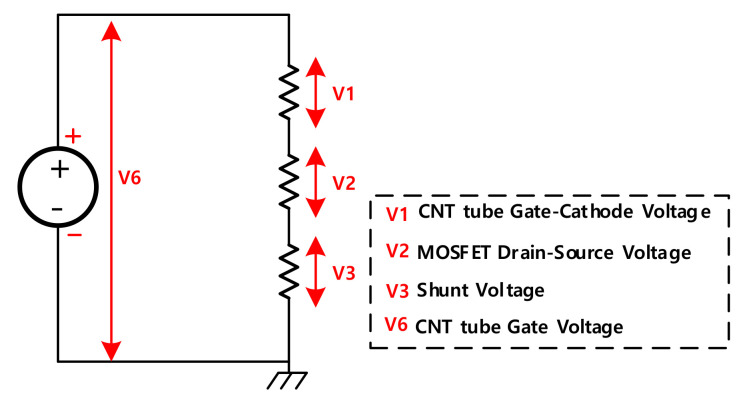
Equivalent circuit for the CNT tube gate-ground to explain the CNT pulsing dynamic.

The presence of high flat is caused by several factors. To explain this phenomenon, the vacuum voltage (Figure 5e) should be emphasized. Figure 6 illustrates that the equivalent closed circuit involves V1, V2, V3, and V6. The exerted voltage of V6 is 2.6 kV. It is divided by three voltages: V1, V2, and V3. Amongst them, V3 has a range from 0V to 10 V. V6 is mostly divided by V1 and V2. According to Figure 5, V1, the vacuum voltage between the CNT tube’s gate and source, takes up 77%, while V2, the drain-source voltage of MOSFET, only takes up 23% even during the off-state of the MOSFET. During the turn off time, the tube current is so low that the vacuum impedance of the gate-cathode is much larger than that of MOSFET. It means even at the off-state of MOSFET, the voltage condition for field emission for the CNT is still satisfied and electron cloud can be formed at the surface of the CNT. 

**Figure 7:**
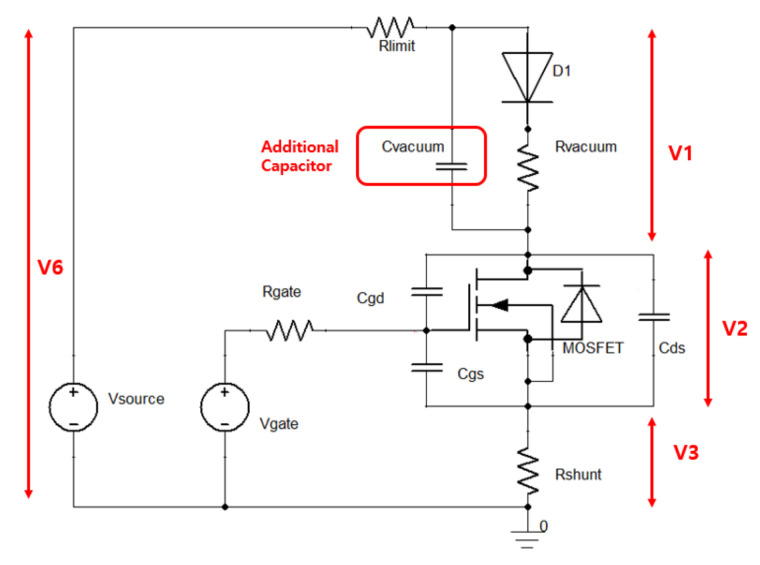
Simulation layout that resembles the dynamic characteristic of CNT pulsing.

**Figure 8:**
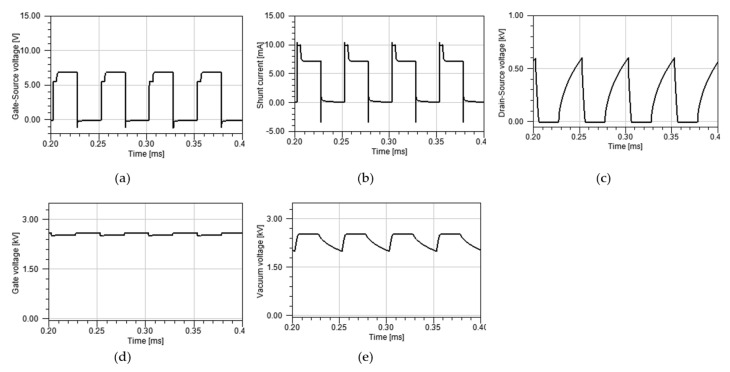
Simulation results with additional capacitor: (**a**) gate-source voltage of MOSFET; (**b**) shunt current; (**c**) drain-source voltage of MOSFET; (**d**) CNT gate voltage; (**e**) vacuum voltage (CNT gate-cathode voltage).

**Figure 9:**
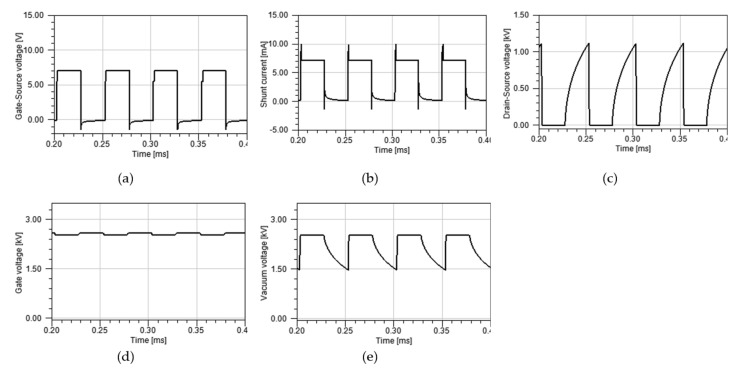
Simulation results without additional capacitor: (**a**) gate-source voltage of MOSFET; (**b**) shunt current; (**c**) drain-source voltage of MOSFET; (**d**) CNT gate voltage; (**e**) vacuum voltage (CNT gate-cathode voltage).

However, the high impedance of MOSFET during the off-state prevents the current from flowing. The field emission occurring during the off-state accumulates free electrons, similar to a capacitor. Once MOSFET is turned on, free electrons can be fired, similar to capacitor discharging. However, according to the discussion on the maximum current issue of the layout in Figure 3, free electrons accumulated during the off-state can only be discharged at the maximum value determined by Equation (2). At the high flat period, the discharged free electrons form a boosted current as shown in Figure 5b and a fundamental current caused by normal field emission is added to the form the high flat. The total charge of electrons caused by field emission during the off-state is given by Equation (3).

(3) $$Q_{\mathrm{FE}}=\int_{t_{\mathrm{off}}}^{t_{\mathrm{on}}}\left(I_{\mathrm{HF}}-I_{\mathrm{LF}}\right)\mathrm{dt}$$

where $Q_{\mathrm{FE}}$ represents the total charge accumulated by field emission during the off-state; $I_{\mathrm{HF}}$ represents the high flat current; $I_{\mathrm{LF}}$ represents the low flat current; and $t_{\mathrm{off}}$ and $t_{\mathrm{on}}$ represent the turn off and turn on time of MOSFET, respectively. Considering the boost current is 2.5 mA (10 mA high flat current and 7.5 mA low flat current) and the high flat period is 4 μs, the calculated total charge is about 100 nC. To further investigate the mechanism of high flat, we built a simulation model as shown in Figure 7. The simulation adds an 80 pF capacitor to model the space charge extraction effect during the off time. Vacuum impedance is modeled with varying impedance. It is closely related to electron density between the tube gate and tube cathode. External conditions such as the tube gate voltage and MOSFET state determine electron densities in the vacuum. The simulation is conducted by ANSYS Simplorer. Figure 8 and Figure 9 each show results of the simulation model with or without the additional capacitor, respectively.

The comparison between Figure 8 and Figure 9 shows the simple capacitor model does simulate charge accumulation during the off-state to some extent. However, the simple capacitor model cannot accurately represent complicated physical phenomenon happening during the off-state. To build more accurate model, further research is required involving the vacuum impedance characteristic, field emission representation, and MOSFET characteristic.

**Figure 10:**
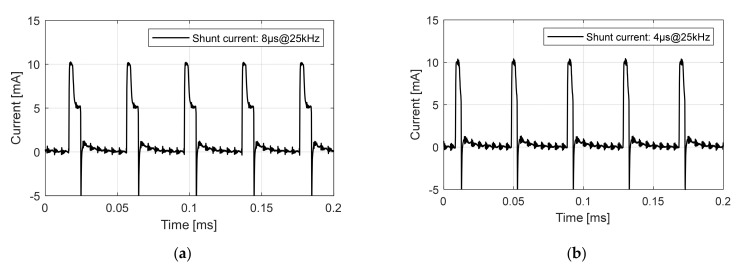
Two types of pulses for dose comparison: (**a**) 8 μs pulse width consists of 4 μs high flat (10 mA) and 4 μs low flat (5 mA); (**b**) 4 μs pulse width of high flat (10 mA).

The explanation about high flat suggests that the boosted current might contribute to the dose. To prove the claim, we conducted dose experiments with two different types of pulses: one with an 8 μs pulse width consisting of a 4 μs high flat (10 mA) and 4 μs low flat (5 mA), and another one with only a 4 μs high flat (10 mA). Both types of pulse had the same maximum current. The frequency of the pulses ranged from 12.5 kHz to 62.5 kHz. The two types of pulses at the 25 kHz frequency are shown in Figure 10. The anode voltage was set to 50 kV, and exposure time was 1 s. The high frequency ripple appearing in Figure 10 is due to noise from the anode power source. 

**Figure 11:**
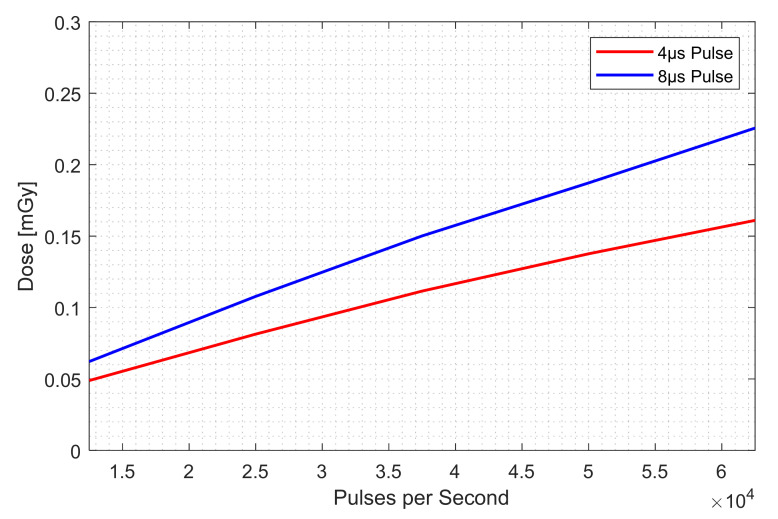
Dose comparison for two types of pulses. If the high flat does not contribute to the dose, the measured dose with 8 μs should be twice that with 4 μs. However, the result shows that the measured dose with an 8 μs pulse is only 34% greater on average than the 4 μs pulse.

Table 1 lists the dose comparison and Figure 11 illustrates the result of Table 1. Both type of pulses showed dose linearity to frequency increase. We can deduce a conclusion about the dose contribution of high flat from the following facts: if the boosted current in high flat does not contribute to the dose, the measured dose with 8 μs should be twice that with 4 μs in Table 1 and Figure 11. However, the result shows that the measured dose with an 8 μs pulse is only 34% greater on average than the 4 μs pulse. The dose difference matches the total current difference in Figure 10, which means that the high flat current or boosted current indeed contributes to the dose.

The high flat effect or combined dynamics brings about two significant benefits: firstly, the boosted current due to free electron accumulation during the off-state of MOSFET can boost a CNT tube’s current capability; secondly, a well calculated circuit according to Equation (2) can determine the maximum current available for an X-ray generator. 

## 5. Conclusions

In recent years, research on CNT-based X-ray tubes has mainly focused on microscopic structures of carbon nanotubes and their effects on performance, or how their manufacturing process associates with stability, longevity, and robustness. The current output performance (free electron extraction performance) is mainly studied by the static characteristic. However, the current output characteristic under a pulsing condition can be complicated. Investigations for both the field emission characteristic and basic MOSFET switching dynamics are required. We refer to it as the combined dynamics of CNT-pulsed X-ray. 

In this work, we studied the combined dynamics of X-ray pulsing using CNT-based tubes. A pulsing current presents two distinct levels of pulses each cycle, which we call “high flat” and “low flat”, respectively. By studying related waveforms and circuit equations, we were able to analyze the mechanism of the high flat, which is the combined dynamics of the field emission, MOSFET characteristic, and modified gate driver circuit. A simple capacitor model was built to simulate the accumulation of charges during the off time. Dose experiments showed that the high flat current does contribute to the dose, which means accumulated charges can be utilized to enhance the CNT current. Furthermore, the maximum current design by the modified gate driver circuit allows us to operate pulses only with a high flat current; the improved pulsing technique can boost a CNT’s current capability and greatly enhance its accuracy.

However, the study of the combined dynamics of CNT pulsing is at the beginning stage. More details need to be analyzed via experiments and data. The relationship between vacuum impedance and drain-source impedance during the off time of the MOSFET may suggest some important information about combined dynamics which could further improve our pulsing technique.

## Figures and Tables

**Table 1:** Dose comparison for two types of pulses.

Pulses per Second	Measured Dose, 4 μs (mGy/s)	Measured Dose, 8 μs (mGy/s)
12,500	0.04889	0.06224
25,000	0.0814	0.1078
37,500	0.1115	0.1501
50,000	0.1376	0.1872
62,500	0.161	0.2256

## Data Availability

The datasets used and/or analyzed during the current study are available from the corresponding author on reasonable request.
